# Rapid determination of polysaccharides in BianTi Soft Extract by spectrophotometry coupled with gas chromatography-mass spectrometry

**DOI:** 10.4103/0973-1296.62895

**Published:** 2010-05-05

**Authors:** Minxia Zheng, Jie Shen, Kai Yang, Songxiang Qian, Sujuan Feng

**Affiliations:** *Traditional Chinese Medical Hospital of Zhejiang Province, No. 54 Youdian Road, Hangzhou, Zhejiang Province - 310 006, P.R. China*; 1 *The First Affiliated Hospital of Medical School of Zhejiang University, No. 79 Qingchun Road, Hangzhou, Zhejiang Province - 310 003, P.R. China*

**Keywords:** BianTi Soft Extract, GC-MS, polysaccharides, quality control, spectrophotometry

## Abstract

A simple approach for the rapid determination of polysaccharides in BianTi Soft Extract using spectrophotometry coupled with gas chromatography-mass spectrometry (GC-MS) was developed. The mixed standard solution composed of D-glucose, D-mannose, galactose and D-xylose in different proportions (1.00: 1.01: 0.12: 0.05) was prepared according to the monosaccharide composition analysis of the polysaccharides by GC-MS. The determination of polysaccharides by UV-Vis spectrophotometer was performed after 35-min color reaction, in which 1 ml 5% phenol and 4 ml sulfate was used. The assay of the method validation has shown that the method was stable, reliable and feasible. Furthermore, the proposed method was successfully applied in the preparation procedure of BianTi Soft Extract, selecting out optimal decoction conditions and suitable decoction container. It suggests that the convenient method could be useful for the quality control of BianTi Soft Extract. Meanwhile, it may be an alternative for polysaccharides determination of other formulations.

## INTRODUCTION

Soft Extract is a well-known traditional medicinal formulation available in most areas of China. Various types of Soft Extract have been used in different prescriptions for different purposes. They can effectively cure sub-health state, resist sickness and keep physical fitness. BianTi Soft Extract is a representative of traditional Chinese prescriptions for the regulation and improvement of immune function. It is composed of classical herbal medicines, including Ginkgo biloba, Rhizoma Dioscoreae, Semen Coicis, Fructus Crataegi, Pericarpium Citri Reticulatae and Fructus Jujubae; polysaccharides have emerged as an important class of bioactive components in this formulation. Recently, polysaccharides have been reported to exhibit a variety of biological activities,[1,2] such as anti-tumor,[3,4] immunostimulation,[5] anti-inflammation,[6,7] anti-complement,[8] anti-oxidation,[9,10] anti-coagulation,[11] anti-fatigue,[12] and enhancement of probiotic bacteria growth.[13]

Polysaccharides are the main bioactive components in BianTi Soft Extract, and the constituents are various and mostly unknown. It is necessary to investigate the content of polysaccharides for the evaluation of quality control of BianTi Soft Extract. Various methods have been developed for the chemical analysis of polysaccharides, including GC-MS, HPLC-MS, CE-DAD, NMR and so on.[14–17] However, most researches are focused on seperation, detection and identification of these compositions, and the methodologies are complicated, abstruse and time-consuming. Also, they are not suitable and convenient for the determination of total polysaccharides.

In view of the above drawbacks, this research was performed with the objective of developing a simple method for polysaccharides determination and applying it into the preparation procedure of BianTi Soft Extract. Moreover, monosaccharide composition of total polysaccharides from this formulation is also studied so that an accurate content of polysaccharides could be obtained.

## MATERIALS AND METHODS

### Reagents and Chemicals

Standard monosaccharides (with a purity of 99%), including D-glucose, galactose, D-xylose and D-mannose, were purchased from Aladdin Reagent Co., Ltd. (Shanghai, China) and National Institute for the Control of Pharmaceutical and Biological Products (Beijing, China),. Sulfate and phenol were of analytical grade and obtained from Sinopharm Chemical Reagent Co., Ltd. (Shanghai, China). Trifluoroacetic acid (CP) and heptane (AR) were also from Sinopharm Chemical Reagent Co., Ltd. Isopropanol and methanol were of HPLC grade and were from Tianjin Shield Company (Tianjin, China). Derivatization reagents (MSTFA and TMCS) and pyridine were of analytical grade and obtained from Sigma Aldrich Fluka (Fluka, U.S.A.). Samples of BianTi Soft Extract were produced by the Pharmacy of Traditional Chinese Medical Hospital of Zhejiang Province.

### Apparatus

Analysis of monosaccharide composition was performed on the Agilent Technologies 6890N Network gas chromatograph equipped with an Agilent Technologies 5973 Network quadrupole mass selective spectrometer and ZB-5MS (Phenomenex) capillary columns (30 m × 0.25 mm I.D., 0.25 μm film thickness). Determination of total polysaccharides was performed on the TU-1901 Double-beam UV-Vis spectrophotometer (Beijing purkinje general instrument Co., Ltd.).

### Preparation of sample solution for GC-MS analysis

Total polysaccharides of BianTi Soft Extract were obtained by dialysis with semi-permeable membrane (MW 3500). The supernatant obtained after total hydrolysis of polysaccharides was incubated with 2 M Trifluoroacetic acid (TFA) at 120°C for 2 h.[18] Then, the supernatant (0.4 ml) was spiked with isopropanol (50 μl) and methanol (50 μl), centrifuged by Thermo Savant SPD121P to dryness. Residues were spiked with pyridine (150 μl) and derivatization reagents (MSTFA: TMCS = 100: 1, v/v, 100 μl). Then derivatization was performed at 70°C for 2 h. Derivatives were diluted with 100 μl heptane and filtrated through the 0.45 μm filter before GC-MS analysis.

### Preparation of sample solution for spectrophotometry analysis

A total of 0.1 ml sample was diluted to 1.5 ml with H_2_O, and then this mixture was spiked with 1 ml phenol (5%) and 4 ml H_2_SO_4_. Color reaction was performed at 30°C for 35 min. 5% Phenol was freshly prepared in our laboratory.

### Preparation of standard solution for polysaccharides determination

Standard stock solution was prepared in the mixed monosaccharide formation according to the GC-MS data of monosaccharide composition. The standard solution was also spiked with 5% phenol (1 ml) and H_2_SO_4_ (4 ml) for color reaction at 30°C for 35 min before spectrophotometer analysis. The blank control was prepared in the same way.

### Assay conditions

GC-MS conditions in this assay are described as follows: Helium was used as the carrier gas at a flow rate of 1.0 ml•min^−1^. The temperature of the injector was 270 °C, and the sample (0.2 μl) was injected in the splitless mode. The column temperature was set at 75°C, and ramped at 8°C•min^−1^ to 125°C, hold for 5 min. Then the temperature was ramped at 7°C•min^−1^ to 220°C, hold for 2 min. Finally, the temperature was ramped to 280°C at a rate of 10°C•min^−1^, hold for 3 min. The tandem quadrupole mass spectrometer was operated in electron impact (EI) mode and full scan monitoring mode (m/z 50-800). The quadrupole temperature was set at 75°C, the source temperature at 230°C, and the electron energy at 70 eV.

Spectrophotometry conditions in the assay were 200-600 nm as its full-scan wavelength, with the slow scanning speed 2 nm as the broadband spectrum and 488 nm as the detection wavelength.

### Method validation

The linearity and range of the analytical procedure were determined by serial dilution of standard stock solution. The calibration curves were constructed by the ratios between the absorbance of serial standard solutions and their concentrations. The precision was evaluated both intra-day and inter-day by the analysis of the standard solution of middle concentration and the data was compared after six consecutive runs (intra-day) and over a six-day period (inter-day). To validate the accuracy of this method, the recovery experiments were performed by adding accurate amounts of standard solution to the samples at low, middle and high concentrations, respectively. Repeatability of the method was examined by six duplicate samples, treated in the same preparation and analyzed under the same condition. The stability experiments of the color reaction were performed on both the standard solution and the sample solution, and then evaluated by the absorbance at six time points during 35 min to 90 min after the reaction.

## RESULTS AND DISCUSSION

### Analysis of monosaccharide composition of the polysaccharides in BianTi Soft Extract

**Figure 1:**
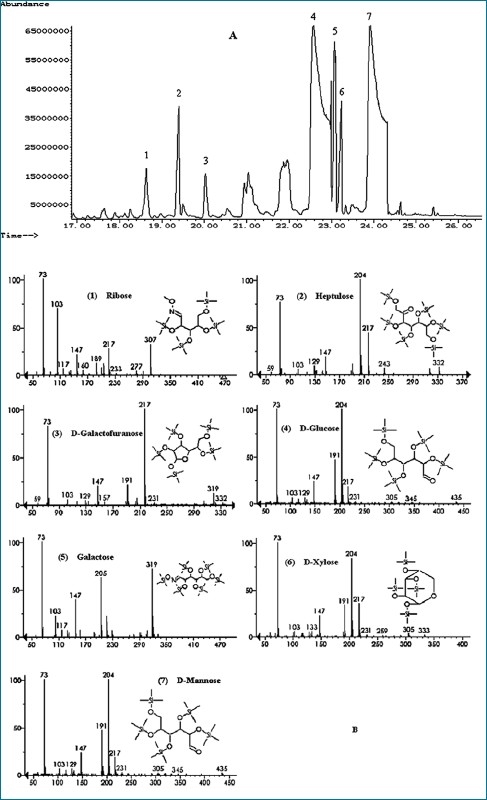
A typical total ion current chromatogram (A) and mass spectrum of the main monosaccharides (B) by GC-MS

The information regarding monosaccharide composition was acquired by GC-MS analysis, with the NIST v1.0.0.12 mass spectra library for the identification of compounds. The total polysaccharides in BianTi Soft Extract were mainly composed of D-glucose, D-mannose, galactose, D-xylose, heptulose, D-ribose and D-galactofuranose [Figures 1A and B]. The relative mass ratios of the monosaccharides were obtained by calculating the ratios between the peak area of each monosaccharide and the peak area of D-glucose. The results are listed in Table 1. Thus, the standard stock solution could be prepared according to the data gained. They were composed of D-glucose, D-mannose, galactose and D-xylose in different proportions (1.00: 1.01: 0.12: 0.05), thereby making the determination of polysaccharides more accurate.

**Table 1:** The relative mass ratio of main monosaccharides hydrolyzed of polysaccharides in BianTi Soft Extract

Peak	t_R_ (min)	Compound	Relative massratio (*n*=6)
1	18.6	D-ribose	0.032
2	19.4	Heptulose	0.046
3	20.0	D-galactofuranose	0.028
4	22.6	D-glucose	1.000
5	23.1	Galactose	0.115
6	23.2	D-xylose	0.052
7	23.9	D-mannose	1.011

### Investigation on the conditions of polysaccharides determination by spectrophotometer

**Figure 2:**
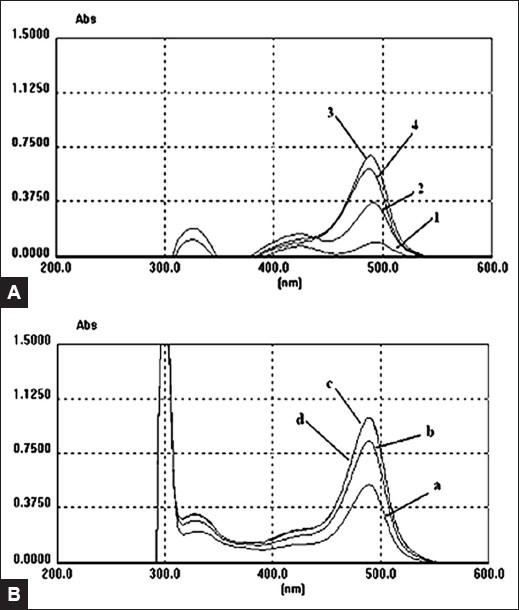
Spectral scans of the standard solution in different color reactions according to various dosages of sulfate (A) and time points (B) (A) dosages of sulfate: (1) 2 ml; (2) 3 ml; (3) 4 ml; (4) 5 ml (B) time points of color reaction: (a) 15 min; (b) 25 min; (c) 35 min; (d) 45 min

Color reaction was the most important procedure in the polysaccharides determination by UV-Vis spectrophotometer. Sulfate and phenol were the reagents for color development in the assay. The dosage of 5% phenol was 1.0 ml and sulfate dosage (2.0, 3.0, 4.0 and 5.0 ml) should be selected for the optimal use of the color reaction. As a result, 4 ml sulfate made the standard solution to exhibit the maximal absorbance in the experiments [Figure 2A]. The time of color reaction (15, 25, 35, 45 min) was also selected and the absorbance after 35 and 45 min were almost the same; moreover, the values were larger than that after 15 and 25 min [Figure 2B]. Therefore, the determination of polysaccharides should be performed after 35 min of the color reaction.

### Method validation

**FIGURE 3:**
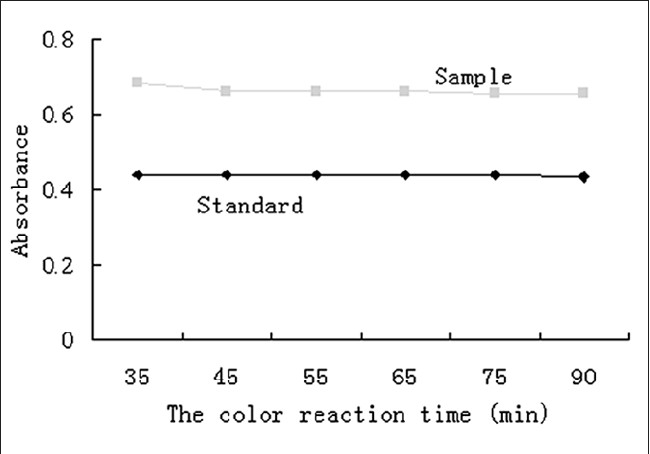
THE RESULT OF COLOR REACTION STABILITY EXPERIMENT IN BOTH STANDARD AND SAMPLE SOLUTION

The mean correlation coefficients (*R*^2^) of the calibration curves, which were higher than 0.9968, showed good linearity of the method in the range of 13.35 - 46.73 μg•ml^−1^ (calculation of D-glucose). The precisions were evaluated as RSD values 0.07% for intra-day assay and 4.81% for inter-day assay, confirming that the method had good precisions. The results of the accuracy test showed that the recovery of low, middle and high concentrations were in the range of 91.1 - 106.2%, within 100 ± 10% to validate this method. The RSD value of reproducibility was below 3.98%, which indicated the good repeatability of the method. The stability test of the color reaction, which included standard solution and sample solution, gave a good result. The RSD values were 0.31% and 1.53% for the standard solution and the sample solution respectively, showing that it was stable for the polysaccharides determination during 35 to 90 min after the reaction [Figure 3]. Therefore, it can be concluded that the method was stable, reliable and feasible.

### Analysis of decoction engineering of BianTi Soft Extract

Addition of general water, decocting time and frequency of decoction were the major factors in herbal decoction procedure. These three factors and three-level orthogonal experiment were analyzed by determining the content of polysaccharides in this assay. The data acquired were used in the ANOVA so that the optimal decoction conditions of BianTi Soft Extract could be obtained. The results are shown in Table 2. The frequency of decoction had a greater impact on the content of polysaccharides than the other two, with the *R* values 5.78>4.53>2.89. Moreover, all the three factors affected the content of polysaccharides if *P*<0.05 while only the frequency of decoction affected if *P*<0.001. It was illustrated that the frequency of decoction had an extremely significant influence on the decoction engineering of BianTi Soft Extract. Considering the production costs and efficiency, it was suggested that three times of decoction, decocting time of 60 min and addition of 8 multiples of water would be the optimal decoction conditions, as evident from decoction engineering analysis.

**Table 2:** The three factors, three-level orthogonal experiment and ANOVA data by the determination of polysaccharides in the decoction procedure of BianTi Soft Extract

	Water added (multiples)	Decocting time (min)	Frequency of decoction	Blank	Polysaccharides content (μg·ml-1)
1	1 (6)	1 (30)	1	1	31.06
2	1 (6)	2 (60)	2	2	38.46
3	1 (6)	3 (90)	3	3	39.20
4	2 (8)	1 (30)	2	3	38.41
5	2 (8)	2 (60)	3	1	40.83
6	2 (8)	3 (90)	1	2	37.54
7	3 (10)	1 (30)	3	2	37.95
8	3 (10)	2 (60)	1	3	35.18
9	3 (10)	3 (90)	2	1	44.26
K1#	108.72	107.42	103.78	116.15	
K2	116.78	114.47	121.13	113.95	
K3	117.39	121.00	117.98	112.79	
R##	2.89	4.53	5.78	1.12	
Mean Square	7.81	15.38	28.48	0.97	
F	8.04	15.84	29.33		
P<0.05	*	*	*		
P<0.001			**		

* There was a significant difference in the level of confidence interval 95%

** There was a significant difference in the level of confidence interval 99.9%

# K^1^=ΣPolysaccharides content at 1 level,

## R= (max(K)-min(K)) / 3

### Selection of appropriate decoction container of BianTi Soft Extract

Porcelain pot, purple casserole and stainless steel pot were often used as decoction containers for Soft Extract in most areas of China. Containers were considered to affect the content of active ingredients in soft extract. In this assay, three kinds of containers including porcelain pot, purple casserole and stainless steel pot were compared with each other to find out the most suitable pot for BianTi Soft Extract. The results indicated that the highest content of polysaccharides was detected with the purple casserole, whose value was 27.43 μg•ml^−1^. However, it was only 10.02 μg•ml^−1^ and 13.15 μg•ml^−1^ with the porcelain pot and the stainless steel pot, respectively. Therefore, it was suggested to use the purple casserole as the decoction container of BianTi Soft Extract.

## CONCLUSION

In this study, an optimized method was established and validated for polysaccharides determination in BianTi Soft Extract. GC-MS-based method was useful and reliable for identifying compounds such as monosaccharides, providing a powerful tool for the analysis of monosaccharide composition. Therefore, the standard monosaccharides solution prepared in different proportions make an accurate determination of polysaccharides. The proposed method was applied in the preparation of BianTi Soft Extract, selecting out the optimal decoction conditions and the suitable decoction container. It suggested that the developed method could be sucessfully applied to the quality control of BianTi Soft Extract. Furthermore, it may be an alternative for polysaccharides determination of other formulations and is of great importance for the rapid determination of unknown compounds using GC-MS coupled with spectrophotometry.
